# 

**Published:** 1965-01

**Authors:** 


					jD 5<j: r- January 1965
3~a.\j.JkHL
SDEXMEDjCUS
1TC k3&-~.,
vr?k:'J3
INCORPORATING THE MEDICAL JOURNAL OF THE SOUTH WEST
Vol 80 (i) No 295

				

